# Development and validation of a nomogram for predicting survival of pulmonary invasive mucinous adenocarcinoma based on surveillance, epidemiology, and end results (SEER) database

**DOI:** 10.1186/s12885-021-07811-x

**Published:** 2021-02-10

**Authors:** Yadong Wang, Jichang Liu, Cuicui Huang, Yukai Zeng, Yong Liu, Jiajun Du

**Affiliations:** 1 Institute of Oncology, Shandong Provincial Hospital, Cheeloo College of Medicine, Shandong University, Jinan, PR China; 2 Department of Thoracic Surgery, Shandong Provincial Hospital, Cheeloo College of Medicine, Shandong University, 324 Jingwu Road, Jinan, 250021 PR China

**Keywords:** Lung cancer, Pulmonary invasive mucinous adenocarcinoma, Nomogram, SEER, Survival

## Abstract

**Background:**

Lung cancer remains the leading cause of cancer death globally. In 2015, the cancer classification guidelines of the World Health Organization were updated. The term “invasive mucinous adenocarcinoma (IMA)” aroused people’s attention, while the clinicopathological factors that may influence survival were unclear.

**Methods:**

Data of IMA patients was downloaded from SEER database. Kaplan-Meier methods and log-rank tests were used to compare the differences in OS and LCSS. The nomogram was developed based on the result of the multivariable analysis. The discrimination and accuracy were tested by Harrell’s concordance index (C-index), receiver operating characteristic (ROC) curve, calibration curve and decision curve analyses (DCA). Integrated discrimination improvement (IDI) index was used to evaluate the clinical efficacy.

**Results:**

According to multivariate analysis, the prognosis of IMAs was associated with age, differentiation grade, TNM stage and treatments. Surgery might be the only way that would improve survival. Area under the curve (AUC) of the training cohort was 0.834and 0.830 for3-and 5-year OS, respectively. AUC for 3-and 5-year LCSS were separately 0.839 and 0.839. The new model was then evaluated by calibration curve, DCA and IDI index.

**Conclusion:**

Based on this study, prognosis of IMAs was systematically reviewed, and a new nomogram was developed and validated. This model helps us understand IMA in depth and provides new ideas for IMA treatment.

**Supplementary Information:**

The online version contains supplementary material available at 10.1186/s12885-021-07811-x.

## Highlights

Pulmonary Invasive mucinous adenocarcinoma (IMA) was a special type of lung adenocarcinoma;

IMA treatment needs more attention.

A nomogram for comprehensive review of IMA treatment and prognosis;

## Background

Lung cancer remains the most commonly diagnosed cancer and the leading cause of cancer death in recent years [1]. Non-small cell lung cancer (NSCLC) accounts for about 85% of lung cancer, among which, lung adenocarcinoma (LUAD) is becoming the main type of NSCLC [2].

In 2015, the cancer classification guidelines of the World Health Organization were updated, and it clarified the new classification of lung tumors. According to the new classification standard, adenocarcinoma was divided into two categories, non-mucinous adenocarcinomas and adenocarcinoma variants. Simultaneously, the term “invasive mucinous adenocarcinoma (IMA)” was proposed to replace the previously named mucinous bronchoalveolar adenocarcinoma [3]. According to the report, IMA accounts for 0.2% of all primary lung cancer [4], and < 2–10% of all lung adenocarcinomas [5], thus it is considered as a relatively rare histologic subtype.

Due to the low incidence, the clinicopathological characteristics and prognosis of IMAs are still unclear and controversial.it was reported that compared to patients with other lung adenocarcinoma subtypes, IMA patients have poor overall survival and progression-free survival times. Meanwhile, IMAs are considered to be diagnosed at an advanced stage of inoperability [6–8]. However, a previous study demonstrated that patients with IMA had comparable overall survival as those with intermediate grade non-mucinous adenocarcinoma (NMA) [9]. Moreover, a study by Yoshizawa et al. showed that the disease-free survival of patients with IMA were between low-grade and high-grade adenocarcinoma [10]. A recent study indicated that the survival curve of IMA patients was between lepidic adenocarcinoma and other adenocarcinoma patients, *and* it found that *~ 70%* of IMAs were either stage I or II at the time of diagnosis [4]. Similarly, Warth et al. found that a better prognosis was available for IMA patients compared with most adenocarcinoma patients [11]. The clinicopathological factors that may influence patient survival were unclear. Thus, it is vital to establish a comprehensive analytic model to accurately estimate the prognosis of each patient.

The nomogram is a commonly viable predictive model for predicting and quantifying the probability of a clinical event, which is of great value for clinical decision-making and risk stratification, especially in cancer patients [12, 13]. However, as far as now, no nomogram has been developed for predicting the survival outcomes of IMA patients. Thus, in the present research, we used the IMAs case data of Surveillance, Epidemiology, and End Results (SEER) database to analyze clinical characteristics and study prognostic factors of IMAs. Furthermore, a nomogram of IMA patients was developed to better predict the cancer-specific survival of patients.

## Methods

### Data source

The SEER database, a cancer incidence registry managed by the National Cancer Institute, includes about 30% of the U.S. population. The data of IMA patients was extracted from SEER database(www.seer.cancer.gov), using SEER*Stat program (version 8.3.5). From the November 2018 submission, patients were collected up to December 2016 to build our cohort following the inclusion criteria: (a)pathological diagnosis was made between 2000 and 2015, (b)the International Classification of Diseases for Oncology-3 (ICD-O-3) histology code 8253/3^d^: Invasive mucinous adenocarcinoma, (c)only one malignant primary tumor. Patients with a diagnosis confirmed by autopsy and/or with incomplete survival data were excluded.

The study variables of patients we extracted and analyzed include: baseline demographics, tumor features, treatments. Baseline demographics include age (≤59y, 60–69y, 70–79y, ≥ 80),gender (Female, Male), race(White, Black, Other), marital status (Divorced, Married, Separated, Single, Windowed), survival time (months) and vital survival status. Tumor features include grade (Well differentiated, Moderately differentiated, Poorly, differentiated/Undifferentiated), laterality (Only one side, Bilateral), SEER–stage (Localized, Regional, Distant), T stage in 8th edition AJCC system (T1, T2, T3, T4), N stage in 8th edition AJCC system (N0, N1, N2/3), M stage in 8th edition AJCC system (M0, M1). Treatments include surgery (No, Yes), radiation (No, Yes), and chemotherapy (No/Unknown, Yes). It had to say that we clarified the 8th TNM stage according to the tumor size, positive lymph nodes and metastasis. While the data of metastasis was not so detailed and we just divided the M stage into M0 and M1 stage.

Overall survival (OS) or lung cancer specific survival (LCSS) was set as the study endpoint. The OS was defined as the time from date of diagnosis to date of death or last contact. The LCSS was defined as the time from date of diagnosis to date of death due to lung cancer.

### Statistical analyses

To make the best use of our data for constructing the predictive model, we use the “=RAND ()” function in the EXCEL software to randomly number the samples and take the first 740 as the training group, and the remaining 184 as the validation group. We used training group to establish the predictive model and to develop the nomogram. The validation group was used to validate the model.

For survival analyses, age at diagnosis, gender, race, marital status, grade, laterality, SEER–stage, T stage in 8th edition AJCC system, N stage in 8th edition AJCC system, M stage in 8th edition AJCC system, surgery, radiation and chemotherapy variables were included. Kaplan-Meier methods and log-rank tests were used to compare the differences in OS and LCSS. The hazard ratio (HR) and corresponding 95% confidential interval (CI) of each potential prognostic variable were estimated by using the univariate and multivariate Cox Proportional Hazard Regression Model. SPSS 25.0 (SPSS, Chicago, IL) was used for the above analysis. Based on the results of the multivariable analysis, a nomogram was developed to provide visualized risk prediction.

Discrimination and calibration were used to evaluate the accuracy of nomogram for predicting visualized risk and survival outcomes. The Harrell’s concordance index (C-index) was used as a measurement tool of discrimination. The Accuracy of calibration was represented by a calibration curve. The reliability of the model was evaluated by Decision curve analyses (DCA). Finally, integrated discrimination improvement (IDI) index was used to compare the clinical applicability between the new model and TNM staging system.

We used R version 3.6.2 (The R Foundation for Statistical Computing, Vienna, Austria) to perform analyses. R project’s packages, like ‘survival’, ‘rms’, ‘foreign’ were used to conduct multivariate COX analysis and draw the nomogram and Calibration plot, and ‘timeROC’, ‘survIDINRI ‘were used to validate the model and perform AUC analysis and IDI analysis. Besides, “stdca.R” was downloaded from Memorial Sloan Kettering Cancer Center(www.mskcc.org) to conduct DCA. In all statistical analyses, a *p* value of < 0.05 was considered significant. This study followed the Declaration of Helsinki for medical research involving human subjects.

## Results

### Patient characteristics

We identified 924 patients diagnosed as invasive mucinous adenocarcinoma (IMA) by immunohistochemistry between 2000 and 2015 (Supplementary Fig. S1). Of all the patients, 740 cases diagnosed were randomly included in univariate analysis.407 of them were included in multivariate Cox regression analysis and used as training cohort of the diagnostic nomogram. Besides, 95 cases of the validation group with complete information were used as validation cohort. All patients had complete information on survival time and cause of death. The 3-year, 5-year overall survival (OS) in training group were 53.7 and 42.8%, respectively. The 3-year, 5-year lung cancer special survival (LCSS) in training cohort were 56.8 and 46.5%, respectively.

The median age was 67.3. The percentage of people whose age ≤ 59, 60–69, 70–79, and ≥ 80 years old was 24.3, 27.0, 34.9, 13.8% respectively. Most patients are white people, about81.1%. The proportion of female are slightly greater than male. Four hundred ninety-four cases have detailed records of pathological grades, among which most (78.3%) are well differentiated. Of all these cases, 528 patients (71.4%) underwent surgery. While the proportion who choose chemotherapy and radiation treatment is significantly lower than surgery, only 3.2 and 28.9% of patients received radiation treatment and chemotherapy, respectively (Table 1).

**Table 1:** Univariate Cox regression for OS and LCSS

Characteristics	Number	OS			LCSS		
		HR	95.0%CI	*p*	HR	95.0%CI	*p*
**Age**	740						
≤ 59	180(24.3%)	1		–	1		–
60–69	200(27.0%)	1.316	0.999–1.733	0.051	1.213	0.899–1.637	0.207
70–79	258(34.9%)	2.034	1.584–2.612	< 0.001	1.714	1.301–2.257	< 0.001
≥ 80	102(13.8%)	3.015	2.239–4.059	< 0.001	2.398	1.722–3.339	< 0.001
**Race**	740						
White	600(81.1%)	1		–	1		–
Black	71(9.6%)	0.944	0.694–1.285	0.714	1.048	0.748–1.467	0.787
Other	69(9.3%)	0.902	0.659–1.235	0.520	1.022	0.730–1.431	0.900
**Gender**	740						
Female	440(59.5%)	1		–	1		–
Male	300(40.5%)	1.302	1.089–1.556	0.004	1.259	1.031–1.539	0.024
**Grade**	494						
Well differentiated	387(78.3%)	1		–	1		–
Moderately differentiated	86(17.4%)	0.987	0.733–1.329	0.932	0.948	0.674–1.333	0.758
Poorly differentiated/Undifferentiated	21(4.3%)	2.127	1.315–3.441	0.002	2.236	1.340–3.732	0.002
**Laterality**	731						
Only one side	701(95.9%)	1		–	1		–
Bilateral	30(4.1%)	4.285	2.946–6.234	< 0.001	5.082	3.482–7.416	< 0.001
**SEER-stage**	720						
Localized	353(49.0%)	1		–	1		–
Regional	172(23.9%)	2.086	1.656–2.628	< 0.001	2.313	1.762–3.038	< 0.001
Distant	195(27.1%)	5.142	4.133–6.396	< 0.001	6.630	5.177–8.491	< 0.001
**Tstage_8th**	591						
T1	209(35.4%)	1		–	1		–
T2	153(25.9%)	1.706	1.266–2.299	< 0.001	1.752	1.193–2.573	0.004
T3	92(15.6%)	2.545	1.844–3.512	< 0.001	3.132	2.111–4.648	< 0.001
T4	137(23.2%)	4.055	3.051–5.389	< 0.001	5.923	4.204–8.343	< 0.001
**Nstage_8th**	684						
N0	587(85.8%)	1		–	1		–
N1	33(4.8%)	2.115	1.438–3.110	< 0.001	2.557	1.708–3.830	< 0.001
N2/3	64(9.4%)	3.537	2.661–4.701	< 0.001	4.080	3.023–5.507	< 0.001
**Mstage_8th**	722						
M0	536(74.2%)	1		–	1		–
M1	186(25.8%)	4.031	3.310–4.910	< 0.001	4.749	3.836–5.879	< 0.001
**Surgery**	740						
No	212(28.6%)	1		–	1		–
Yes	528(71.4%)	0.235	0.194–0.285	< 0.001	0.210	0.170–0.260	< 0.001
**Radiation**	740						
No	716(96.8%)	1		–	1		–
Yes	24(3.2%)	1.880	1.213–2.915	0.005	2.024	1.275–3.213	0.003
**Chemotherapy**	740						
No/Unknown	526(71.1%)	1		–	1		–
Yes	214(28.9%)	2.321	1.926–2.797	< 0.001	2.829	2.308–3.468	< 0.001
**Marital_status**	715						
Divorced	58(8.1%)	1		–	1		–
Married	451(63.1%)	0.805	0.582–1.113	0.189	0.761	0.533–1.086	0.132
Separated	12(1.7%)	0.816	0.366–1.819	0.619	0.887	0.373–2.110	0.786
Single	77(10.8%)	0.816	0.539–1.235	0.336	0.727	0.457–1.158	0.180
Widowed	117(16.4%)	1.041	0.720–1.505	0.831	0.969	0.644–1.457	0.879

### Univariate and multivariate cox proportional hazard analysis

We conducted univariable and multivariable analysis to identify the prognostic factors associated with survival of IMA patients in the training cohort.

**Fig. 1:**
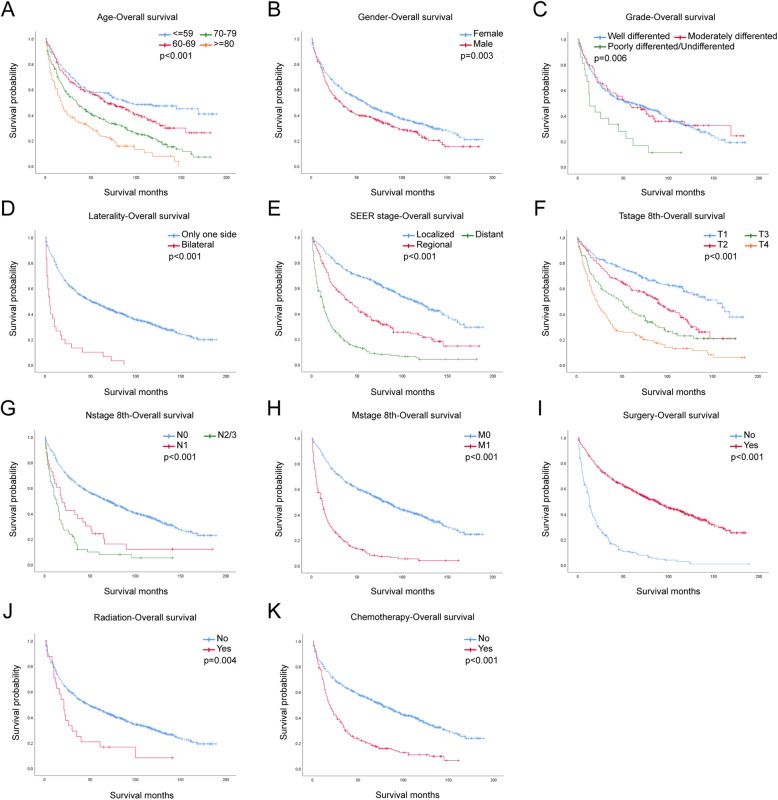
OS for IMA patients stratified by (**a**) Age, *p* < 0.001; **b** Gender, *p* = 0.003; **c** Grade, *p* = 0.006; **d** Laterality, *p* < 0.001; **e** SEER stage, *p* < 0.001; **f** T-stage, *p* < 0.001; **g** N-stage, *p* < 0.001; **h** M-stage, *p* < 0.001; **i** Surgery, *p* < 0.001; **j** Radiation, *p* = 0.004; **k** Chemotherapy *p* < 0.001

**Fig. 2:**
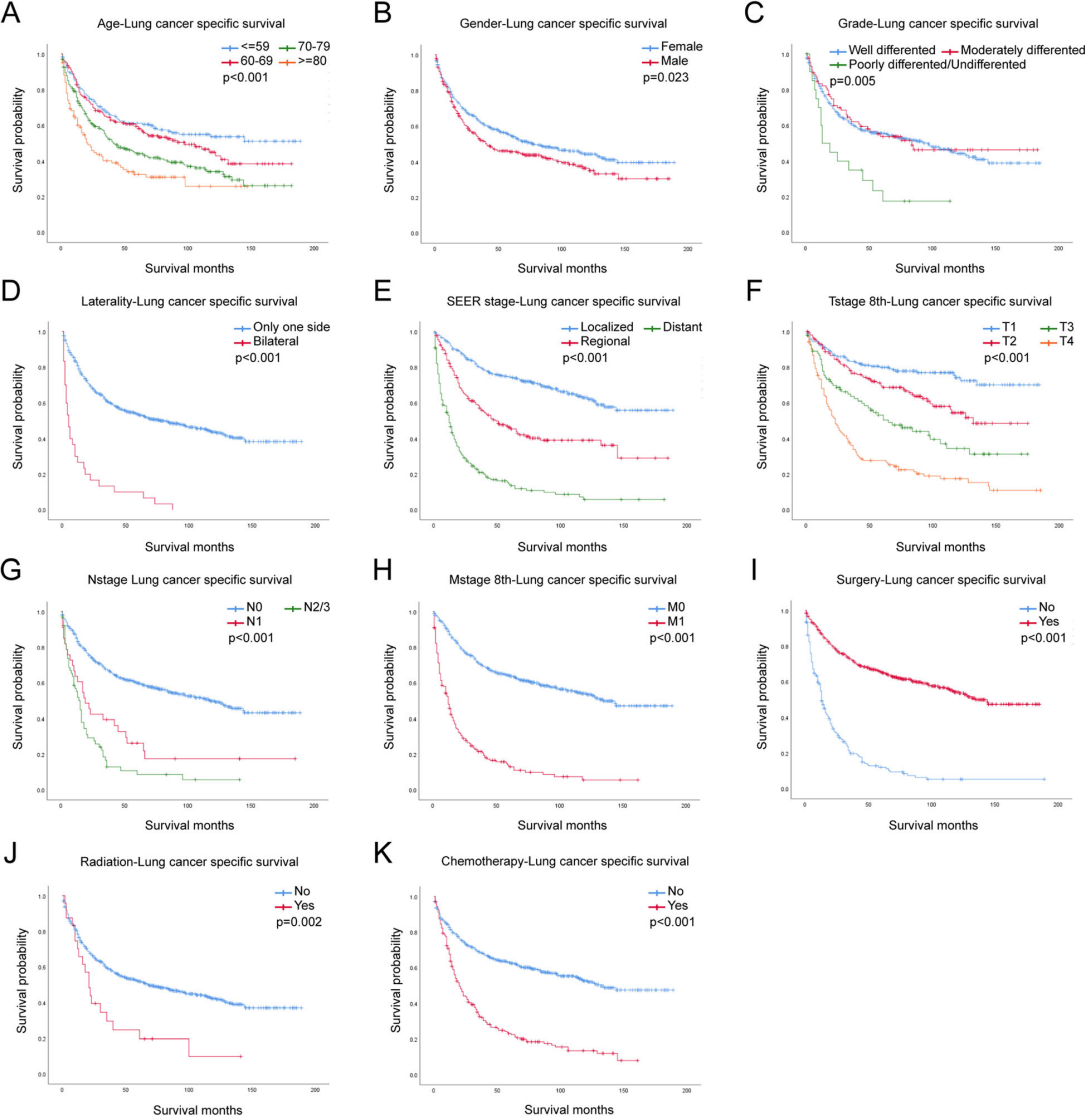
LCSS for IMA patients stratified by (**a**) Age, *p* < 0.001; **b** Gender, *p* = 0.023; **c** Grade, *p* = 0.005; **d** Laterality, *p* < 0.001; **e** SEER stage, *p* < 0.001; **f** T-stage, *p* < 0.001; **g** N-stage, *p* < 0.001; **h** M-stage, *p* < 0.001; **i** Surgery, *p* < 0.001; **j** Radiation, *p* = 0.002; **k** Chemotherapy *p* < 0.001

In the univariate analysis, older age, male, poorly differentiated grade, bilateral laterality, higher TNM stage, no surgery, radiation treatment and chemotherapy predicted worse OS and LCSS (Fig. 1, Fig. 2). However, race and marital status had no significant effect on OS or LCSS (Supplementary Fig. S2, Fig. S3). Results of univariate Cox regression for OS and LCSS were stated in Table1. All variables were statistically significant (*p* < 0.05) and included into the multivariate analysis.

In the multivariate analysis of both OS and LCSS, variables including age, grade, TNM stage and treatments including surgery, radiation treatment and chemotherapy were all statistically significant. However, there was a slight difference between OS and LCSS about gender. In multivariate analysis, gender had no statistically significant effect on the patient ‘s OS (*p* = 0.143) and LCSS (*p* = 0.592).

Results of multivariate Cox regression for OS and LCSS were stated in Table2.According to multivariate analysis, the outcomes were improved in patients with younger age, well differentiated stage, lower TNM stage and appropriate therapies.

### Development and validation of a prognostic nomogram

**Fig. 3:**
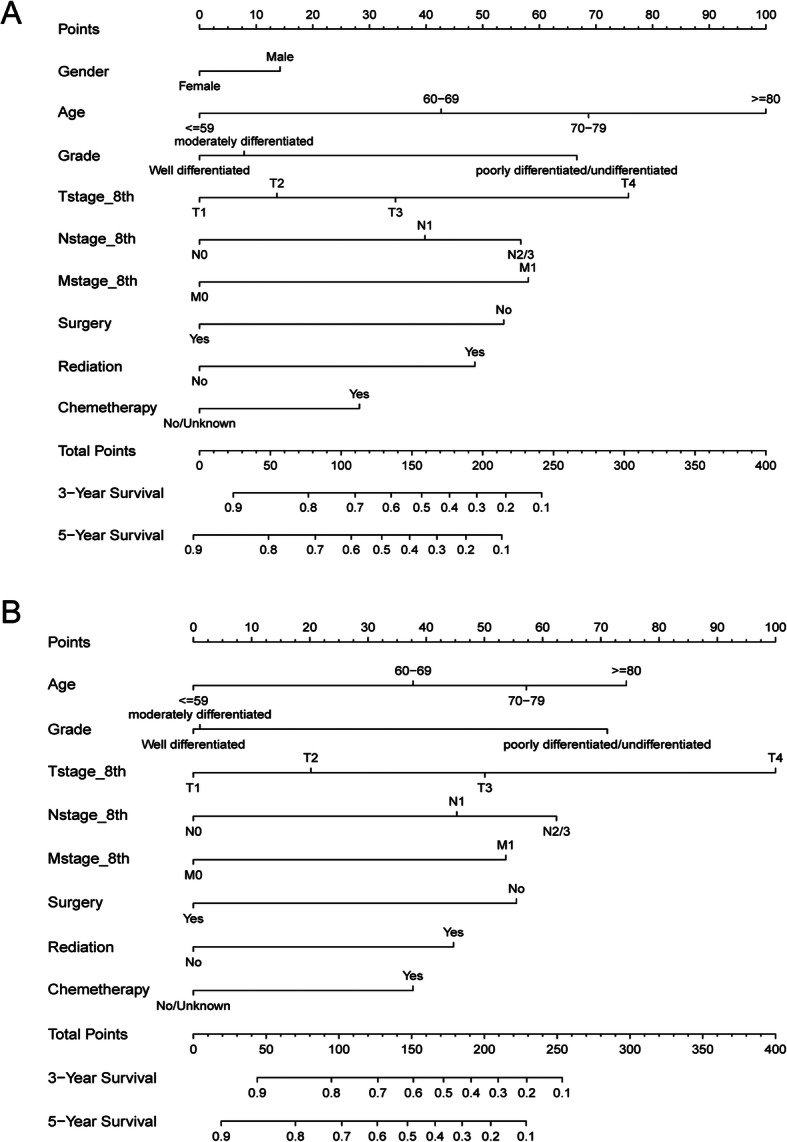
Prognostic nomogram predicting the probability of 3- and 5-year (**a**) overall survival (OS) and (**b**) lung cancer specific survival (LCSS). Each subtype within these significant independent variables was assigned a score on the point scale. The total score projected to the bottom scale

Factors with *p* < 0.2 in the multivariate analysis were used to develop a nomogram to calculate the 3- and 5-year OS or LCSS probabilities (Fig. 3). The nomogram showed that age was the most predominant contributor to the OS followed by T stage which played a more important role for LCSS. Each subtype within these significant independent variables was assigned a score on the point scale. The total score projected to the bottom scale representing the probabilities of 3- and 5-year OS or LCSS.

**Fig. 4:**
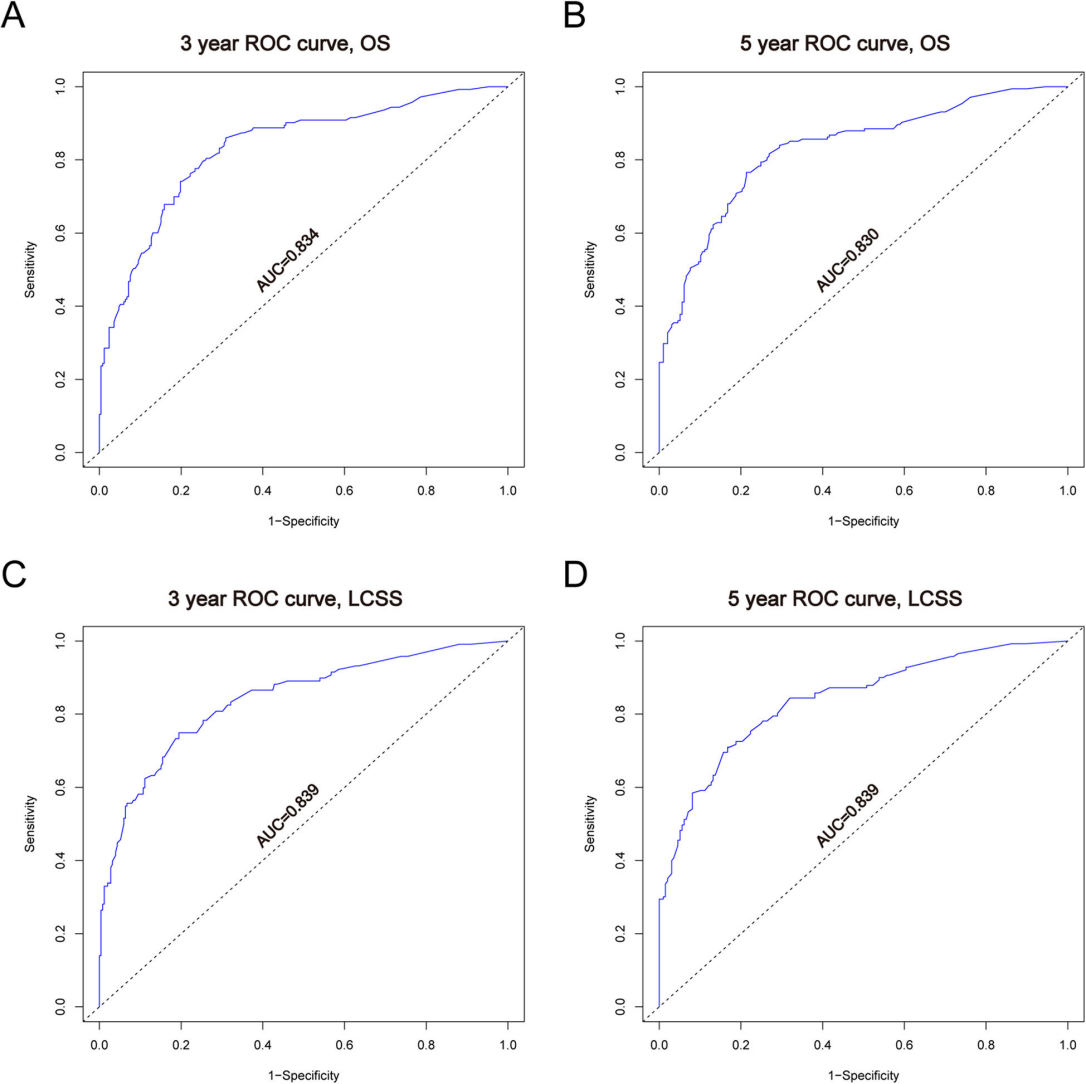
**a**. **b** ROC curves for 3- and 5-year OS based on the nomogram. The AUC was 0.834 and 0.830, respectively; **c**. **d** ROC curves for 3- and 5-year LCSS. The AUC was 0.839 and 0.839, respectively

**Fig. 5:**
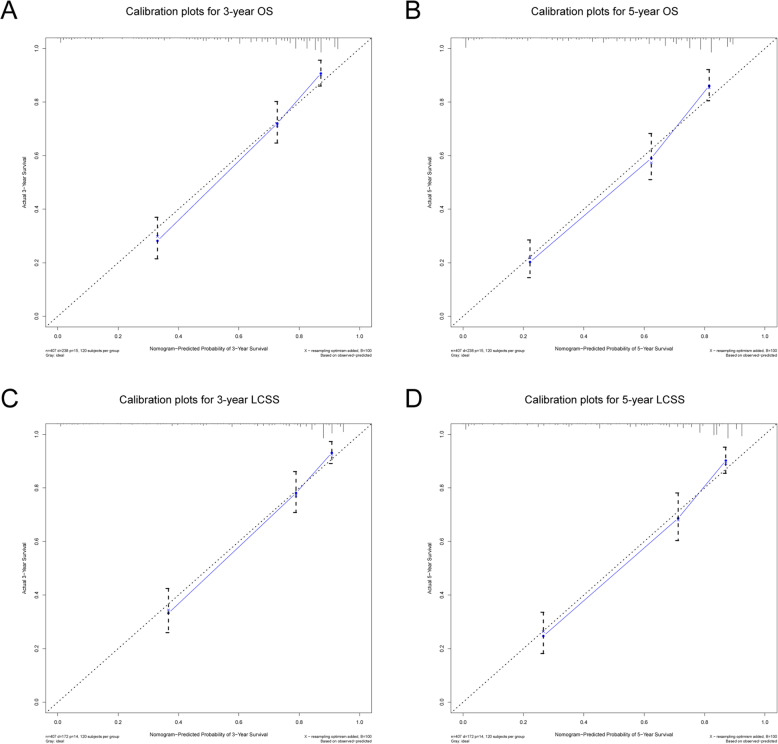
**a**. **b** Calibration plots for 3- and 5-year OS in the training cohort; **c**. **d** Calibration plots for 3- and 5-year LCSS in the training cohort

In the training cohort, C-index was 0.766 (95%CI: 0.737–0.795) for OS, 0.791 (95%CI: 0.760–0.822) for LCSS. C-index was 0.757 (95%CI: 0.694–0.820) for OS, 0.778 (95%CI: 0.707–0.849) for LCSS in the validation cohort. We tested the nomogram by internal receiver operating characteristic (ROC) curves in the training cohort. The area under the curve (AUC) was 0.834(95%CI: 0.791–0.876) and 0.830(95%CI: 0.789–0.872) for 3- and 5-year OS, respectively, with 0.839(95%CI: 0.794–0.884) and 0.839(95%CI: 0.796–0.882) for 3- and 5-year LCSS (Fig. 4). Figure 5 showed the calibration plots of the nomogram. These indicated that the new prediction model had a great performance for IMAs.

**Fig. 6:**
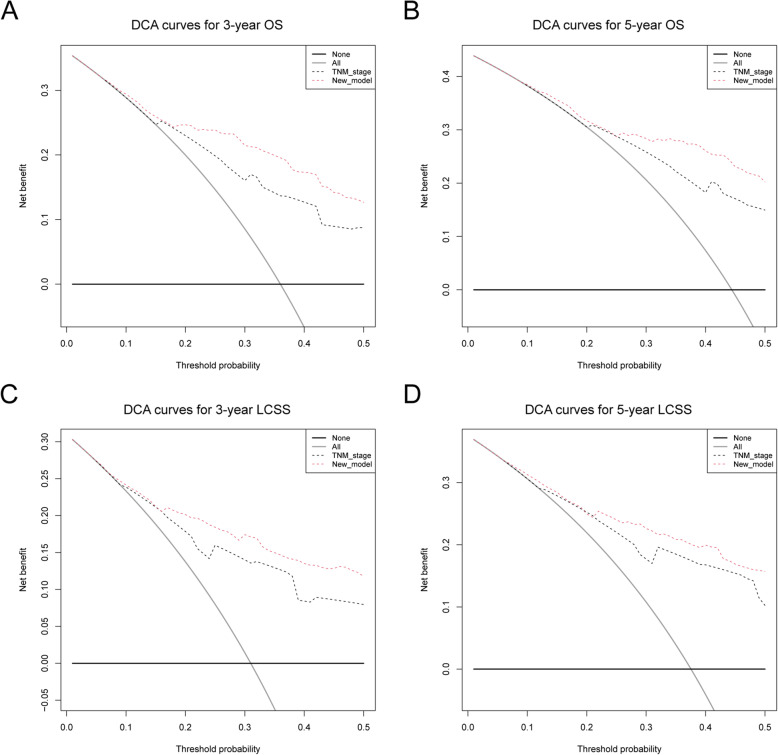
**a**. **b** DCA curves for 3- and 5-year OS comparing this new model with 8th TNM staging system; **c**. **d** DCA curves for 3- and 5-year LCSS comparing this new model with 8th TNM staging system

DCA curve was used to compare the clinical usability and benefits between the nomogram with the 8th edition AJCC TNM staging system (Fig. 5). Discrimination improvement was confirmed by an integrated discrimination index (IDI) with 0.098(95%CI: 0.059–0.149), 0.105(95%CI: 0.068–0.162) for 3−/5- year OS and 0.105(95%CI: 0.068–0.162), 0.105(95%CI: 0.068–0.162) for 3−/5- year LCSS (Fig. 6).

This new model was validated in the validation cohort and the accuracy was proved (Supplementary Fig. S4, S5). Characteristics of the training and validation cohort were presented in Table S1. These results indicated that in clinical application, this new model was better than the 8th edition AJCC TNM staging system.

## Discussion

Lung cancer remains the first cause of oncological death, and in recent years [1], LUAD is becoming more and more frequent [2]. Although the proportion of IMAs in LUAD is relatively low, it was believed that people with IMA had worse prognosis.

IMAs was different from other LUAD, characterized by goblet or columnar tumor cells with abundant intracytoplasmic mucin and basally located nuclei. In some cases, IMAs showed the mixture of different pathological types [14, 15]. IMAs have special genetic signatures. Studies found that many genes unexpectedly enriched in mucin-producing gastrointestinal, pancreatic, and breast cancer showed significant differences in IMAs, including FOXA3, SPDEF, etc. [16]. And there was evidence that B7-H4 expressed in IMAs, which was considered as a therapeutic target for immune checkpoint therapy [16]. Besides, Kadota K et al. and Righi L et al. found that IMA was connected with KRAS mutation [17, 18], while NRG1 fusion looked frequent in IMAs even without KRAS mutations [19–21]. These unique pathological features may affect pathological diagnosis.

In recent years, there have been relatively few studies on systematic reviews of IMAs treatment. Therefore, we decided to constructed a nomogram to predict the prognosis for IMAs and helped to provided new sight for treatment.

In this research, patients diagnosed with IMA was included into our analysis. There are just over 1000 patients, and we included 407 patients with complete clinical information into the training group (Supplementary Fig. S1). These patients had a reasonable age distribution, and most had received surgery.

In univariate analysis, gender, age, differentiation grade, TNM stage, and treatments including surgery, radiation, chemotherapy were all related to IMAs progression (Table 1, Fig. 1, Fig. 2). Surgery treatment would decrease the HR, while radiation treatment and chemotherapy would not.

Gender and age were reported that they had a significantly effect on survival and the results were all similar, that elderly patient patients and male patients faced a higher risk [1, 22, 23]. It was harder to understand why gender would affect the survival than age. It may be because of hormones, smoking, stress, work, etc., but these are still hypothesis, and the reasons still need to be explored in depth.

We conducted multivariate analysis using these significant variables in univariate analysis (Table 2). Except for laterality and gender, all the factors were statistically significant for OS. This result verified that older age, poorly differentiated grade, bilateral laterality, higher TNM stage, no surgery, radiation and chemotherapy were independent prognostic factors and improved the HR.

**Table 2:** Multivariate Cox regression for OS and LCSS

Characteristics	OS			LCSS		
	HR	95.0%CI	*p*	HR	95.0%CI	*p*
Gender						
Female	1		–	1		–
Male	1.223	0.934–1.601	0.143	1.091	0.793–1.503	0.592
Age						
≤ 59	1		–	1		–
60–69	1.830	1.223–2.740	0.003	1.742	1.097–2.768	0.019
70–79	2.646	1.813–3.862	< 0.001	2.312	1.493–3.581	< 0.001
≥ 80	4.121	2.555–6.648	< 0.001	3.007	1.695–5.336	< 0.001
Laterality						
Only one side	1		–	1		–
Bilateral	0.994	0.386–2.562	0.991	1.074	0.408–2.827	0.886
Grade						
Well differentiated	1		–			
Moderately differentiated	1.118	0.787–1.586	0.534	1.003	0.661–1.522	0.989
Poorly differentiated/Undifferentiated	2.569	1.483–4.450	< 0.001	2.793	1.531–5.093	< 0.001
Tstage_8th						
T1	1		–	1		–
T2	1.214	0.828–1.778	0.320	1.351	0.819–2.229	0.238
T3	1.633	1.051–2.538	0.029	2.063	1.193–3.566	0.010
T4	2.925	1.996–4.287	< 0.001	4.298	2.671–6.917	< 0.001
Nstage_8th						
N0	1		–	1		–
N1	1.756	1.044–2.954	0.034	1.936	1.100–3.409	0.022
N2/3	2.232	1.337–3.726	0.002	2.524	1.465–4.348	< 0.001
Mstage_8th						
M0	1		–	1		–
M1	2.278	1.436–3.612	< 0.001	2.202	1.327–3.653	0.002
Surgery						
No	1		–	1		–
Yes	0.467	0.305–0.715	< 0.001	0.452	0.277–0.737	0.001
Radiation						
No	1		–	1		–
Yes	1.991	1.149–3.449	0.001	1.931	1.061–3.514	0.031
Chemotherapy						
No/Unknown	1		–	1		–
Yes	1.492	1.062–2.096	0.021	1.730	1.193–2.509	0.004

IMAs were mainly found in lower lobes and presented with multifocal consolidation and lung-to-lung or pleural metastasis [24]. However, the data of primary site was not complete and we could only analyze the effect of tumor metastasis. It was clear that the number of positive lymph nodes and metastasis would significantly influence the survival time of patients.

Many researches indicated tumor size and invasive size might be the independent factor influencing the prognosis of IMAs [9, 25],but it was quite difficult to clarify the invasive size. In this research, we analyzed the connection between T stage and tumor survival and found that a higher T stage was related to a worse survival.

It was reported that on-TKI chemotherapy was used in many advanced-stage IMA patients, while the OS seemed no improvement [14, 24]. More than 70% patients received surgery。 Consistent with previous reports, chemotherapy does not promote prognosis, and surgery looked like the only treatment that would improve survival [26]. Therefore, for patients with a clear diagnosis of IMA, we still recommended surgical treatment as the first choice. But the effect of surgery combined with chemoradiotherapy or radiation treatment remains to be seen.

We plotted nomograms based on independent prognostic factors suggested in multiple factors (Fig. 3). For OS, age was the main factor that influenced prognosis, and T stage for LCSS. The accuracy of this model was measured via ROC curves and calibration plots. The larger the AUC, the higher the accuracy of the model. The training cohort AUC was 0.834(95%CI: 0.791–0.876) and 0.830(95%CI: 0.789–0.872) for 3- and 5-year OS, respectively, with 0.839(95%CI: 0.794–0.884) and 0.839(95%CI: 0.796–0.882) for 3- and 5-year LCSS (Fig. 4). All these results indicated that this model had a pretty good accuracy for the prediction of IMAs’ survival. At the same time, the Calibration curve also verifies the predictive ability of the model for the overall sample (Fig. 5).

In order to evaluate the model’s clinical value, we compared the model with 8th edition AJCC TNM staging system. IDI was 0.098(95%CI: 0.059–0.149), 0.105(95%CI: 0.068–0.162) for 3−/5- year OS and 0.105(95%CI: 0.068–0.162), 0.105(95%CI: 0.068–0.162) for 3−/5- year LCSS (Fig. 6). The results showed that this model had a better predictive value for IMAs than TNM staging system (Fig. 6).

In this research, we classify bronchioloalveolar carcinoma (BAC) between 2000 to 2015 as IMA. As we can see, the incidence increased in 2001 and 2005, which might be related to the update of WHO classification [3]. While after 2011, the incidence decreased gradually (Supplementary Fig. S6). We did not find any evidence why the incidence of IMA decreased and we analyzed that this might due to that the IASLC/ERS/ATS new recommendations made some detailed modifications and gave many stricter requirements which divided mucinous BAC into Invasive Mucinous Adenocarcinoma, mucinous Minimally Invasive Adenocarcinoma and mucinous Adenocarcinoma in situ. Therefore, the incidence of IMA decreased although IMA account for the majority of BAC as reported.

This retrospective research summarized the characteristics of IMA and construct a prediction model based on SEER database. The comprehensive clinical information of the SEER database provided great support for the study. However, there are many limitations that must be considered. IMA is difficult to diagnose till now, and many patients are classified as “adenocarcinoma” without specific pathological types. At the same time, the number of patients with a clear diagnosis of IMA in the databases around us is also very small, and we have not been able to verify the accuracy of this model in other databases. However, this model comprehensively evaluates the clinical characteristics and treatment, and provides ideas for improving the prognosis of IMA.

## Conclusions

In conclusion, we conducted an analysis of prognosis of IMA based on a large population-based group from SEER database. Prognosis of IMAs was reviewed, and a new nomogram was developed and validated. Then we elucidated the factors that affect IMA prognosis, including gender, age, TNM staging, grade of tumor differentiation and treatments. This model allows us to have a deeper understanding of IMA. At the same time, given its analysis of cancer treatment, it is expected to provide new ideas for IMA treatment.

## Supplementary Information

**Additional file 1: Figure S1.** Flow sheet of eligible patients included in the study. **Figure S2.** OS for IMA patients stratified by (A) Race, *p* = 0.772; (B) Marital status, *p* = 0.226. **Figure S3.** LCSS for IMA patients stratified by (A) Race, *p* = 0.959; (B) Marital status, *p* = 0.267. **Figure S4**. (A. B) ROC curves for 3- and 5-year OS based on the validation cohort data. The AUC was 0.813 and 0.840, respectively; (C. D) ROC curves for 3- and 5-year LCSS. The AUC was 0.836 and 0.857, respectively. **Figure S5.** (A. B) Calibration plots for 3- and 5-year OS in the validation cohort; (C. D) Calibration plots for 3- and 5-year LCSS in the validation cohort. **Figure S6.** Frequency of IMAs in SEER database between 2000 and 2015.

**Additional file 2: Table S1.** Characteristics of the training and validation cohort.

## Data Availability

All data used in this study is available at www.seer.cancer.gov.

## Abbreviations

IMA: Invasive mucinous adenocarcinoma
OS: Overall Survival
LCSS: Lung cancer specific survival
C-index: Concordance index
ROC: Receiver operating characteristic
AUC: Area under the curve
DCA: Decision curve analyses
IDI: Integrated discrimination improvement
CI: Confidence interval
HR: Hazard ratio
SEER database: Surveillance, Epidemiology, and End Results database
