# Supplementary material for: Development and validation of a nomogram for predicting survival of pulmonary invasive mucinous adenocarcinoma based on surveillance, epidemiology, and end results (SEER) database
Source: BMC Cancer. 2021 Feb 10;21:148. doi: 10.1186/s12885-021-07811-x (PMC7877040; doi:10.1186/s12885-021-07811-x)
Supplement: Supplementary file 1 — Additional file 1: Figure S1. Flow sheet of eligible patients included in the study. Figure S2. OS for IMA patients stratified by (A) Race, p = 0.772; (B) Marital status, p = 0.226. Figure S3. LCSS for IMA patients stratified by (A) Race, p = 0.959; (B) Marital status, p = 0.267. Figure S4. (A. B) ROC curves for 3- and 5-year OS based on the validation cohort data. The AUC was 0.813 and 0.840, respectively; (C. D) ROC curves for 3- and 5-year LCSS. The AUC was 0.836 and 0.857, respectively. Figure S5. (A. B) Calibration plots for 3- and 5-year OS in the validation cohort; (C. D) Calibration plots for 3- and 5-year LCSS in the validation cohort. Figure S6. Frequency of IMAs in SEER database between 2000 and 2015. [file 12885_2021_7811_MOESM1_ESM.docx]

**Supplementary materials**


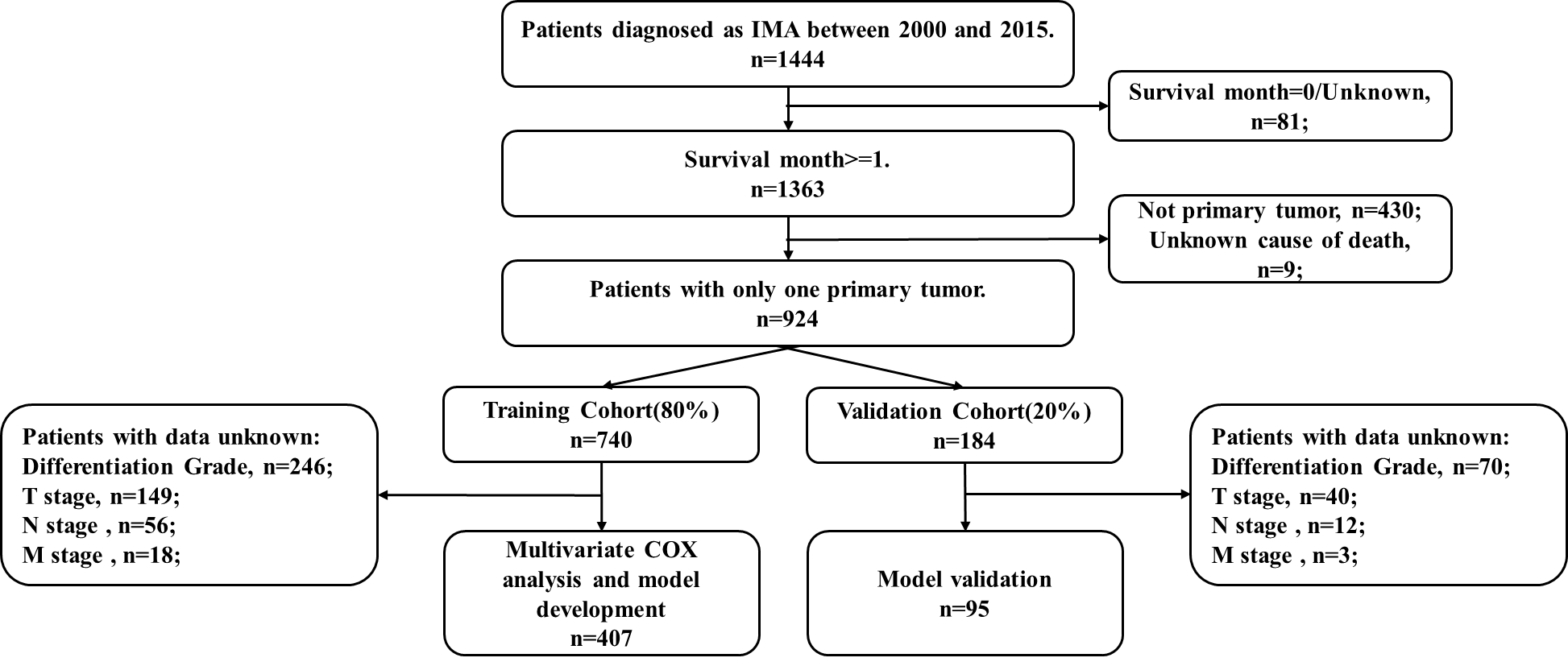


Figure S1. Flow sheet of eligible patients included in the study.


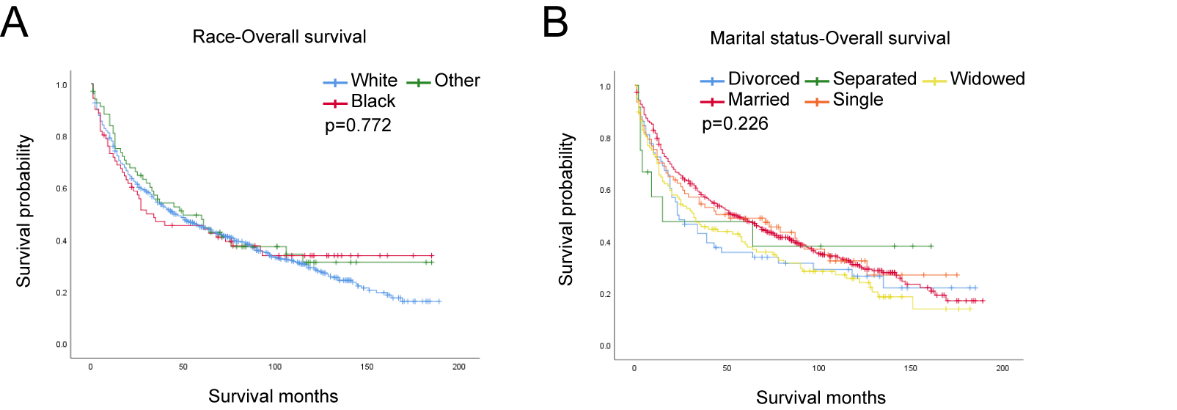


Figure S2. OS for IMA patients stratified by (A) Race, p=0.772; (B) Marital status, p=0.226.


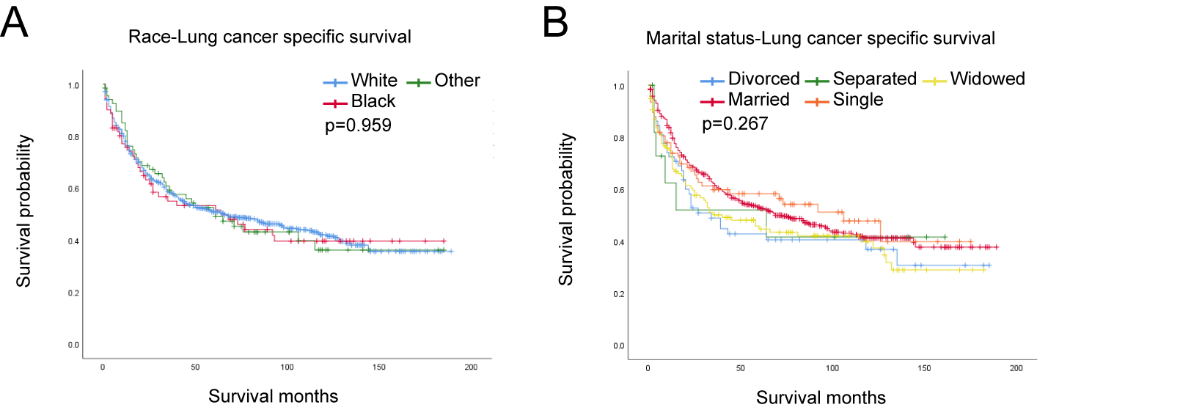


Figure S3. LCSS for IMA patients stratified by (A) Race, p=0.959; (B) Marital status, p=0.267.


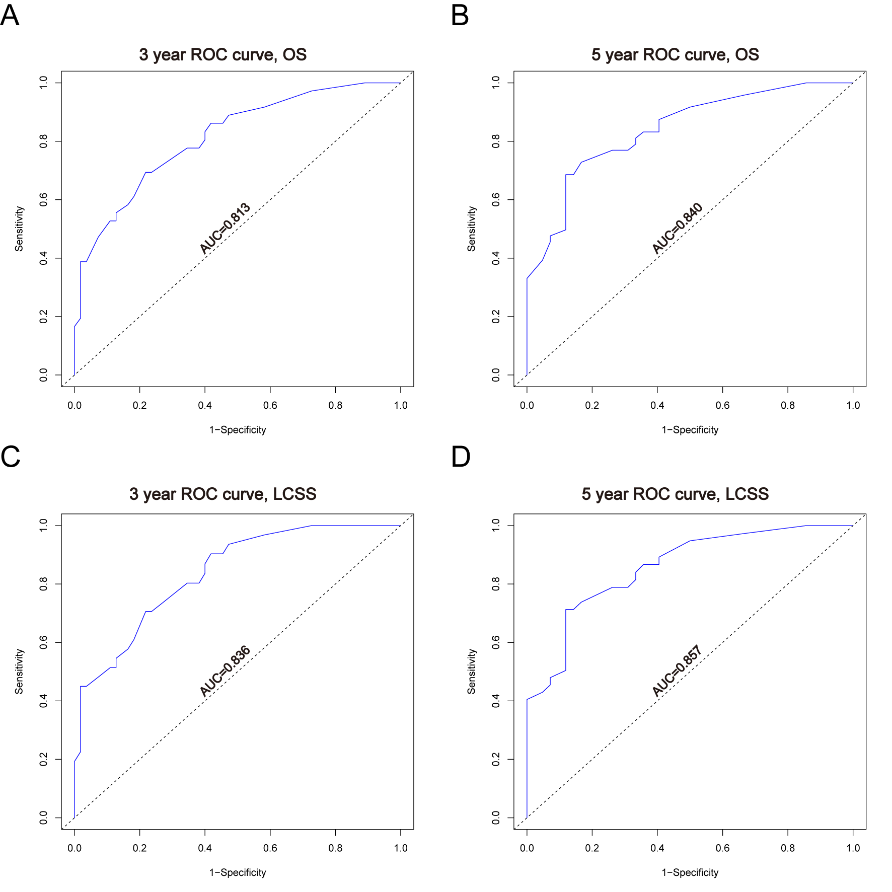


Figure S4. (A. B) ROC curves for 3- and 5-year OS based on the validation cohort data. The AUC was 0.813 and 0.840, respectively; (C. D) ROC curves for 3- and 5-year LCSS. The AUC was 0.836 and 0.857, respectively.


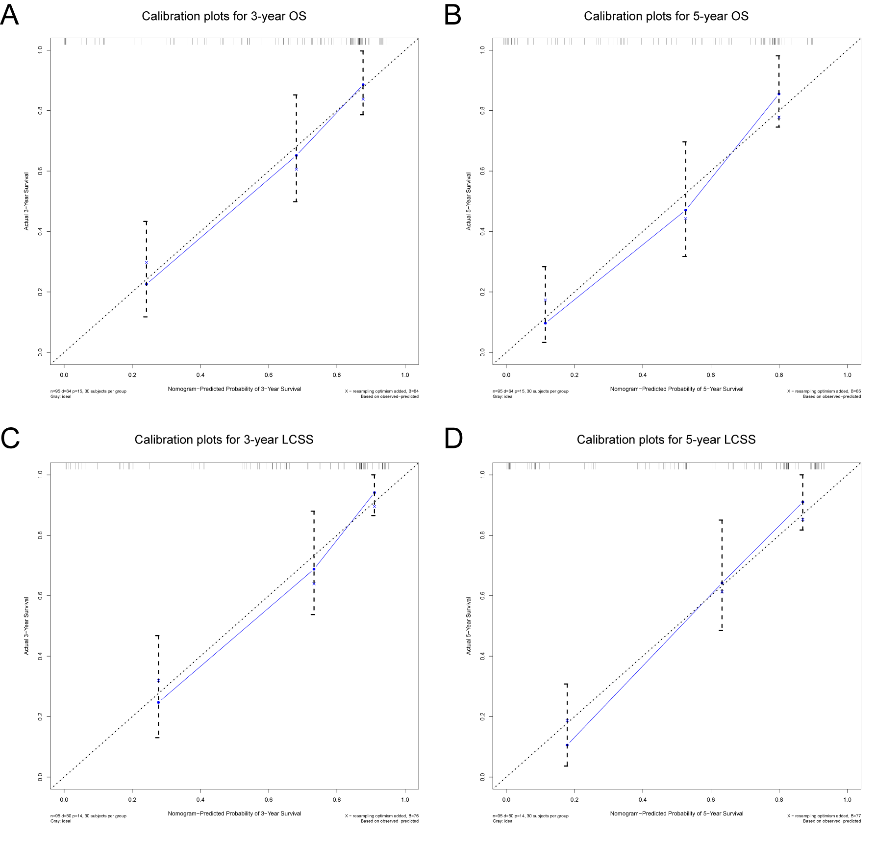


Figure S5. (A. B) Calibration plots for 3- and 5-year OS in the validation cohort; (C. D) Calibration plots for 3- and 5-year LCSS in the validation cohort.


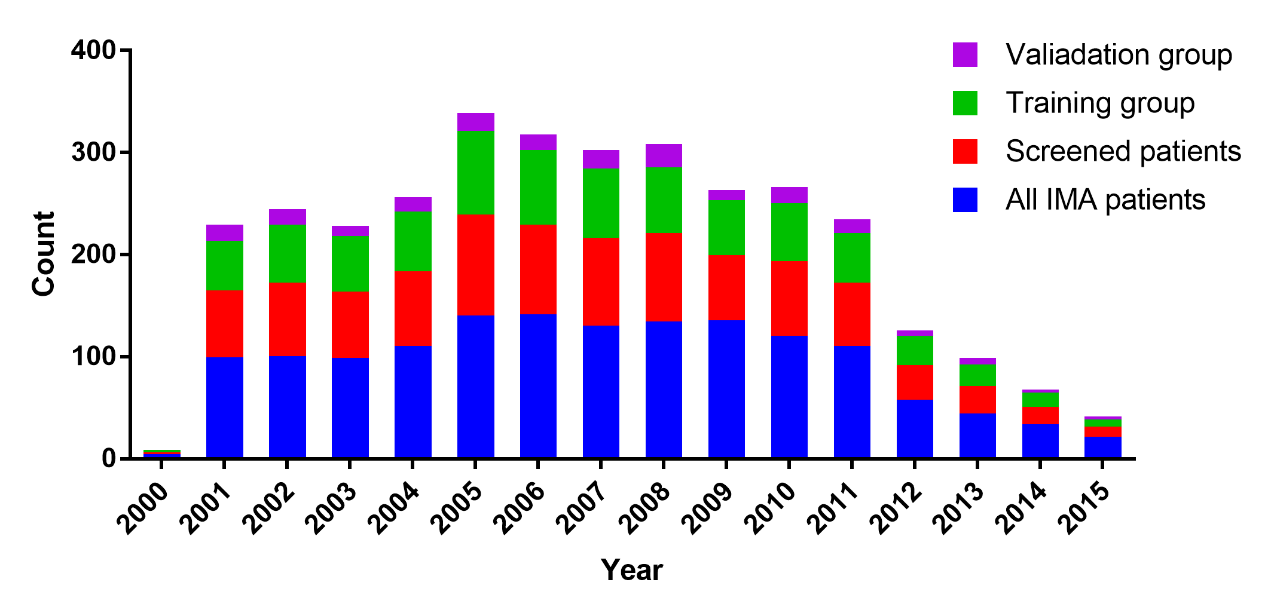


Figure S6. Frequency of IMAs in SEER database between 2000 and 2015.
