# Supplementary material for: Development and validation of a nomogram for predicting survival of pulmonary invasive mucinous adenocarcinoma based on surveillance, epidemiology, and end results (SEER) database
Source: BMC Cancer. 2021 Feb 10;21:148. doi: 10.1186/s12885-021-07811-x (PMC7877040; doi:10.1186/s12885-021-07811-x)
Supplement: Supplementary file 2 — Additional file 2: Table S1. Characteristics of the training and validation cohort. [file 12885_2021_7811_MOESM2_ESM.docx]

**Supplementary Table 1.** Characteristics of the training and validation cohort.

| **Characteristics** | **Training** | | **Validation** | | *p* |
| --- | --- | --- | --- | --- | --- |
|  | Frequency | Percent | Frequency | Percent |  |
| **Age** |  |  |  |  |  |
| ≤59 | 108 | 26.5 | 25 | 26.3 | 0.998 |
| 60-69 | 121 | 29.7 | 29 | 30.5 |  |
| 70-79 | 133 | 32.7 | 31 | 32.6 |  |
| ≥80 | 45 | 11.1 | 10 | 10.5 |  |
| Total | 407 | 100.0 | 95 | 100.0 |  |
| **Gender** |  |  |  |  |  |
| Female | 244 | 60.0 | 59 | 62.1 | 0.699 |
| Male | 163 | 40.0 | 36 | 37.9 |  |
| Total | 407 | 100.0 | 95 | 100.0 |  |
| **Grade** |  |  |  |  |  |
| Well differentiated | 313 | 76.9 | 74 | 77.9 | 0.858 |
| Moderately differentiated | 76 | 18.7 | 18 | 18.9 |  |
| Poorly/Undifferentiated | 18 | 4.4 | 3 | 3.2 |  |
| Total | 407 | 100.0 | 95 | 100.0 |  |
| **Tstage_8th** |  |  |  |  |  |
| T1 | 135 | 33.2 | 34 | 35.8 | 0.315 |
| T2 | 116 | 28.5 | 20 | 21.1 |  |
| T3 | 61 | 15.0 | 20 | 21.1 |  |
| T4 | 95 | 23.3 | 21 | 22.1 |  |
| Total | 407 | 100.0 | 95 | 100.0 |  |
| **Nstage_8th** |  |  |  |  |  |
| N0 | 358 | 88.0 | 80 | 84.2 | 0.401 |
| N1 | 19 | 4.7 | 4 | 4.2 |  |
| N2/3 | 30 | 7.4 | 11 | 11.6 |  |
| Total | 407 | 100.0 | 95 | 100.0 |  |
| **Mstage_8th** |  |  |  |  |  |
| M0 | 359 | 88.2 | 81 | 85.3 | 0.432 |
| M1 | 48 | 11.8 | 14 | 14.7 |  |
| Total | 407 | 100.0 | 95 | 100.0 |  |
| **Surgery** |  |  |  |  |  |
| No | 50 | 12.3 | 14 | 14.7 | 0.519 |
| Yes | 357 | 87.7 | 81 | 85.3 |  |
| Total | 407 | 100.0 | 95 | 100.0 |  |
| **Radiation** |  |  |  |  |  |
| No | 390 | 95.8 | 92 | 96.8 | 0.647 |
| Yes | 17 | 4.2 | 3 | 3.2 |  |
| Total | 407 | 100.0 | 95 | 100.0 |  |
| **Chemotherapy** |  |  |  |  |  |
| No | 312 | 76.7 | 70 | 73.7 | 0.541 |
| Yes | 95 | 23.3 | 25 | 26.3 |  |
| Total | 407 | 100.0 | 95 | 100.0 |  |
